# Almond Skin Extracts Abrogate HSV-1 Replication by Blocking Virus Binding to the Cell

**DOI:** 10.3390/v9070178

**Published:** 2017-07-10

**Authors:** Carlo Bisignano, Giuseppina Mandalari, Antonella Smeriglio, Domenico Trombetta, Maria Musarra Pizzo, Rosamaria Pennisi, Maria Teresa Sciortino

**Affiliations:** Department of Chemical Biological Pharmaceutical and Environmental Sciences, University of Messina, Messina 98166, Italy; cbisignano@unime.it (C.B.); gmandalari@unime.it (G.M.); asmeriglio@unime.it (A.S.); dtrombetta@unime.it (D.T.); marymusarra@live.it (M.M.P.); rpennisi@unime.it (R.P.)

**Keywords:** herpes simplex virus 1, flavonoids, antiviral activity, binding mechanisms, almond skin

## Abstract

The aim of the present research was to determine the effect of almond skin extracts on herpes simplex virus 1 (HSV-1) replication. Drug-resistant strains of HSV frequently develop following therapeutic treatment. Therefore, the discovery of novel anti-HSV drugs deserves great effort. Here, we tested both natural (NS) and blanched (BS) polyphenols-rich almond skin extracts against HSV-1. HPLC analysis showed that the prevalent compounds in NS and BS extracts contributing to their antioxidant activity were quercetin, epicatechin and catechin. Results of cell viability indicated that NS and BS extracts were not toxic to cultured Vero cells. Furthermore, NS extracts were more potent inhibitors of HSV-1 than BS extracts, and this trend was in agreement with different concentrations of flavonoids. The plaque forming assay, Western blot and real-time PCR were used to demonstrate that NS extracts were able to block the production of infectious HSV-1 particles. In addition, the viral binding assay demonstrated that NS extracts inhibited HSV-1 adsorption to Vero cells. Our conclusion is that natural products from almond skin extracts are an extraordinary source of antiviral agents and provide a novel treatment against HSV-1 infections.

## 1. Introduction

Almond skins (also referred as almond bran) represent 4–8% of the total shelled almond weight. In peeled almonds, they are industrially removed by hot water blanching, which results in a substantial loss of bioactive compounds in the blanch water [1]. Specifically, almond skins are rich in dietary fiber, consisting of intrinsic plant cell-wall polysaccharides, which exert beneficial effects in the large bowel through colonic fermentation [2,3]. Furthermore, almond skins represent a source of phenolic compounds, such as flavonols, flavanones and flavan-3-ols, whose health-promoting properties depend on their bioaccessibility in the upper gastrointestinal tract [4]. We have previously demonstrated that polyphenols from almond skin are bioaccessible in the gastric and small intestinal compartment in vitro, and their release is affected by the food matrix, where the skin is incorporated [5]. Although the amount of polyphenols and the antioxidant properties of almond skins are affected by industrial processing [6], several reports have indicated modifications of the human urinary metabolome after intake of almond skin polyphenols [7,8]. The activity of almond polyphenols for scavenging free radicals and inducing quinone reductase has also been documented, with a dose-dependent effect related to the extraction methodologies and interaction with vitamins [9]. We have previously demonstrated neuroprotective effects of almond skins in an experimental spinal cord injury model and a reduction of oxidative stress and inflammation in an experimental model of inflammatory bowel disease after treatment with natural almond skins [10,11]. The antimicrobial potential of polyphenols extracted from almond skins has also been evaluated: both natural and blanched almond skins were active against the Gram-positive strains of *Listeria monocytogenes* and *Staphylococcus aureus*, whereas the Gram-negative *Salmonella enterica* was sensitive to natural almond skin [12,13]. The antiviral effect of natural almond skins has been demonstrated against herpes simplex virus type 2 (HSV-2) in peripheral blood mononuclear cells [14,15]. However, the data were obtained in a semi-permissive cell system to HSV-1 and HSV-2 [16,17]. Herpes simplex virus represents a persistent human pathogen that resides in infected hosts for their lifetime. Indeed, following primary infection, the virus can undergo a lytic infection in epithelial cells and a latent infection in sensory neurons [18]. Since HSV infections are often subclinical, the infection is widely becoming one of the world’s most prevalent sexually-transmitted infections (STIs). In particular, the incidence and severity of infections have increased over the past few decades due to the increasing number of immunocompromised patients, including HIV seropositive patients [19]. Standard treatment of symptomatic HSV infections are based on nucleoside analogues, such as acyclovir (ACV), valacyclovir (VCV) and famciclovir (FAM), which target viral DNA polymerase. These drugs can be used to treat primary or recurrent infections. However, the resistance of HSV to acyclovir has become an important clinical problem, especially among immunocompromised patients exposed to long-term therapy [20]. The discovery of new sources of natural anti-HSV agents that prevent the establishment of infection by inhibiting virus entry and/or viral replication has become fundamental. Based on this, in the present study, we wanted to examine the antiviral effect of natural (NS) and blanched (BS) almond skin extracts against herpes simplex virus type 1 (HSV-1) using a fully-permissive cellular model, such as Vero. We evaluated the cellular proliferation index on Vero cells treated with both NS and BS for the antiviral activity by the reduction plaques assay. In addition, NS was enrolled to evaluate whether almond treatment interferes with the production of new viral progeny. Therefore, a real-time PCR was performed, and the results were confirmed, evaluating the treatment-mediated challenge in the viral antigens expression whose synthesis occurs in a cascade fashion. Finally, a binding assay and infections with VP26GFP HSV-1 expressing a GFP-tagged capsid protein VP26 were carried out, leading to identifying the molecular antiviral mechanism of the NS on the HSV-1 replicative cycle. 

## 2. Materials and Methods

### 2.1. Chemicals

All solvents used (methanol, acetonitrile, acetic acid, water and ethyl acetate) were HPLC-grade and were purchased from Merck (Darmstadt, Germany). Protocatechuic acid, 4-hydroxybenzoic acid, vanillic acid, chlorogenic acid, trans-*p*-coumaric acid, eriodictyol-7-*O*-glucoside, eriodictyol, naringenin, naringenin-7-*O*-glucoside, kaempferol-3-*O*-rutinoside, kaempferol-3-*O*-glucoside, isorhamnetin-3-*O*-glucoside, quercetin, kaempferol, isorhamnetin, quercetin-3-*O*-rutinoside, quercetin-3-*O*-galactoside, quercetin-3-*O*-glucoside, isorhamnetin-3-*O*-rutinoside, catechin and epicatechin were purchased from Extrasynthese (Genay, France). Other chemicals were of analytical grade. 

### 2.2. Sample Origin

Natural almonds (Maisie Jane’s, Chico, CA, USA) were kindly provided by the Almond Board of California and stored in the dark. Natural almond skins (NS) were cryo-peeled with liquid nitrogen by repeated cycles of freeze-thawing, manually removed [2] and crushed in the presence of liquid nitrogen using an analytical mill (Model A 11 BASIC IKA). Blanched almond skins (BS), produced by ABCO Laboratories (Almond skins powder 1912) by soaking brown skin almonds in near boiling water (90–100 °C) for 2–3 min, were supplied by the Almond Board of California.

### 2.3. Sample Preparation

NS and BS were processed according to Mandalari et al. [2]. Briefly, 5 g of NS or BS were extracted three times with *n*-hexane (10 mL) for 6 h under constant agitation in order to remove the lipid fraction. After filtration, the residue was mixed with 50 mL of methanol/HCl 0.1% (*v*/*v*) mixture and sonicated for 15 min. The sample was centrifuged (5000× *g*, 10 min, 4 °C) and the pellet extracted two more times. The methanol fractions were combined and concentrated to dryness by a rotary evaporator; the residue was dissolved in 20 mL of MilliQ water and extracted four times with 20 mL of ethyl acetate. The organic phases were combined and dried with Na_2_SO_4_ for 20 min. The yields of the residues from NS and BS were 7.92% and 6.72%, respectively. The dried extracts of NS and BS were dissolved in methanol (stock solution 10 mg/mL) for HPLC, total phenols and radical scavenging analyses and in DMSO (stock solution 100 mg/mL) for antiviral assays.

### 2.4. Total Phenols Determination

The total phenol content of NS and BS was determined colorimetrically using the Folin-Ciocalteu method as described previously [21]. The total phenol content was expressed as mg of gallic acid equivalents (GAE)/100 g of NS and BS. Each assay was performed in triplicate at least three times.

### 2.5. Radical Scavenging Activity

The anti-radical activity of NS and BS was determined using the stable 2,2-diphenyl-1-picrylhydrazyl radical (DPPH^•^) and the procedure previously described [22]. Results were expressed as mg of extract needed to scavenge 50 µmols of the initial DPPH concentration (SE_50_).

### 2.6. Determination of Polyphenolic Profile

The determination of polyphenolic compounds was performed by reverse phase high performance liquid chromatography coupled with diode array and fluorescence detectors (RP-HPLC-DAD-FLU) as described previously with some modifications [23]. An Agilent high performance liquid chromatography system (1100 series, Agilent, Santa Clara, CA, USA) equipped with a UV-Vis photodiode-array detector (DAD) (G1315) and a fluorescence detector (G1321) coupled with a control system (G1323) equipped with an LC pump (G1312) and an auto-injector (G1313) was used. Separation was performed on a 5-μm ODS3 reversed-phase Prodigy column (250 mm × 4.6 mm; Phenomenex, Torrance, CA, USA). A gradient elution consisting of Solvent A (water/acetic acid, 97:2, *v*/*v*) and Solvent B (water/acetonitrile/acetic acid, 73/25/2, *v*/*v*/*v*) was applied at a flow rate of 1.0 mL/min as follows: 0 min 100% A, 0–55 min 20% A, 55–57 min 10% A, 57–90 min 0% A, 90–95 min 100% A and equilibrated 15 min for a total run time of 110 min. Samples were filtered before through a 0.22-μm PTFE filter, and the injection volume was 20 μL. The detection conditions were set at 270 nm for phenolic acids and flavanones, 330 and 370 nm for flavonols, isoflavones and flavones. The UV spectra of the different compounds were recorded from 190–400 nm. The wavelengths used for fluorescence detection of flavan-3-ols were λ_ex_: 276 nm and λ_em_: 316 nm respectively. Data acquisition was performed using ChemStation software (version A.10.01, Agilent, Santa Clara, CA, USA).

The polyphenol identification was made according to UV-Vis spectra and retention time with respect to commercially-available standards. Quantification was carried out by external standard calibration curves.

### 2.7. Cells Culture and Virus

VERO cell lines (American Type Culture Collection) were propagated in minimal essential medium (EMEM), supplemented with 6% fetal bovine serum (FBS) (Lonza, Belgium) at 37 °C under 5% CO_2_. The prototype HSV-1 (F) strain was kindly provided by Dr. Bernard Roizman (University of Chicago, IL, USA). The quantification of viral DNA was carried out by using TaqMan real-time PCR; the forward and reverse primers (For-59 CATCACCGACCCGGAGAGGGAC; Rev-59 GGGCCAGGCGCTTGTTGGTGTA) were designed on the HSV-1 genome, as well as the TaqMan probe (-59 6FAMCCGCCGAACTGAGCAGACACCCGCGC-TAMRA), where 6FAM is 6-carboxyfluorescein and TAMRA is 6-carboxytetramethylrhodamine. Viral stocks were propagated and then titered in Vero cells. The viral infection was performed by exposure of Vero cells to the HSV-1 virus at the multiplicity of infection MOI of 1. After the infection, the supernatant was replaced with fresh culture medium and collected at the established times of the experimental design. VP26GFP-HSV-1 virus expressing a GFP tagged capsid protein VP26 was propagated and titered in Vero cells as described previously [24].

### 2.8. Cell Proliferation Assay

Vero cells were grown in wells of 96-well plates and treated with three different concentrations of almond extracts (0.4 mg/mL, 0.2 mg/mL and 0.1 mg/mL). The cell viability was determined with a cytotoxicity bioassay kit (Lonza Group Ltd., Basel, Switzerland) according to the manufacturer’s instructions. The GloMax^®^ Multi Microplate Luminometer (Promega Corporation, 2800 Woods Hollow Road, Madison, WI, USA) in combination with the ViaLight^™^ plus cell proliferation and cytotoxicity bioassay kit quantified the emitted light intensity related to ATP degradation. The measured luminescence value was converted to the cell proliferation index (%) according to the following Equation (1):

(1) $$\mathrm{Cell}\;\mathrm{viability}\;\%=(\frac{A-B}{C-B})\%$$

where *A* denotes the average of treated sample, *B* represents background luminescence and *C* represents the average of untreated samples.

### 2.9. Plaque Reduction Assay

The antiviral activity was evaluated by plaque reduction assay. The virus was diluted to yield 60 plaques/100 µL. Samples were inoculated on monolayers of Vero cells in 24-well dishes and incubated for 1 h at 37 °C. After the incubation time, the inoculum was removed, and the monolayers were covered with Dulbecco’s Modified Eagle’s Medium containing 0.8% methylcellulose in the presence of NS and BS extracts at three different concentrations (0.4 mg/mL, 0.2 mg/mL and 0.1 mg/mL), separately. After 3 days, the cells were fixed, stained with crystal violet and visualized with an inverted microscope (Leica DMIL, Nuβloch, Germany) for plaque detection. The data were analyzed as the means of triplicates ± SD for each dilution.

### 2.10. Protein Extraction and Immunoblot Analysis

Vero cells were mock infected or infected with HSV-1 (F) at MOI 1 at 37 °C with gentle shaking. After the incubation time, the inoculum was removed, and the cells were incubated at 37 °C, under 5% CO_2_, for 24 h in infection medium, which included different concentrations of NS and BS extracts (0.4 mg/mL). After 24 h, cells were subjected to protein extraction for Western blot analysis. The protein samples were resolved by SDS-PAGE and transferred to the nitrocellulose membrane. The HSV-1 antigens expression was analyzed by Western blotting. The membranes were incubated with a primary antibody against viral antigens; ICP0 (sc-56985) from Santa Cruz Biotechnology (Santa Cruz, CA, USA); ICP8 and anti-US11 were kindly provided by Dr. Bernard Roizman. Horseradish peroxidase anti-rabbit and anti-mouse antibodies were from Santa Cruz Biotechnology. GAPDH (sc-32233) was purchased from Santa Cruz and used as the loading control. The chemiluminescent signals were detected with SuperSignal^™^ West Pico Chemiluminescent Substrate (Thermo Fisher Scientific, Waltham, MA, USA).

### 2.11. DNA Extraction and Quantitative Real-Time PCR

Vero cells were infected with HSV-1 (F) at MOI 1 for 1 h at 37 °C. After incubation, the inoculum was replaced by fresh growth medium with either NS or BS extract (0.4 mg/mL), separately. Samples were collected and resuspended in of TRIzol reagent (Invitrogen, Carlsbad, CA, USA) and used for DNA extraction, according to the manufacturer’s instructions. The DNA was precipitated, and quantification was carried out as previously described [25]. Briefly real-time PCR was carried out in a 25-µL reaction mixture containing 1 µL of DNA preparation, 0.5 µM each forward and reverse primer (For-59 CATCACCGACCCGGAGAGGGAC; Rev-59 GGGCCAGGCGCTTGTTGGTGTA), 300 nM TaqMan probe (59 6FAMCCGCCGAACTGAGCAGACACCCGCGC-TAMRA, where 6FAM is 6-carboxyfluorescein and TAMRA is 6-carboxytetramethylrhodamine), and 12.5 µL of Maxima Probe qPCR Master Mix (2X) (Maxima Probe qPCR; Fermentas Life Sciences, Burlington, ON, Canada). The amplification was carried out with the aid of a Cepheid SmartCycler II System (Cepheid Europe, Maurens-Scopont, France) under the following conditions: incubation for 10 min at 95 °C, followed by 40 cycles of 30 s at 95 °C, 30 s at 55 °C and 30 s at 72 °C, with a final cycle of 5 min at 72 °C. The relative quantitation of HSV-1 DNA was generated by comparative *C*_t_ method.

### 2.12. The Binding Assay

The binding assay was performed at 4 °C, a temperature that allows the virus to bind to cellular receptors, but not enter the cells. Thus, the only mechanism that a potential inhibitor can disrupt in this assay is virus binding to the cell. Viral suspension was incubated with 0.4 mg/mL NS for 1 h prior to performing the assay and incubated in ice. Vero cells were plated in 6-well plates and allowed to reach confluence. The plates were then removed from the incubator and left at room temperature for about 15 min, after which they were incubated at 4 °C. The cold inoculum contained NS in the presence of viruses or control viruses were used to infect the cells. Plates were then incubated at 4 °C for 1 h to allow the virus to bind to the cells. The unbound viruses were removed by washing the plates 3 times with cold PBS. Plates were overlaid with complete medium and incubated at 37 °C. The cytopathic effect (ECP) was detected by: (i) direct observation of ECP by inverted microscope; (ii) relative quantitation of HSV-1 DNA using real-time PCR. Separately, the binding assay was performed by using VP26-HSV-1 virus and measurement of the auto-fluorescence of VP26-tagged protein. To detect the autofluorescence of the VP26-tagged virus, samples obtained from the binding assay were collected 24 h p.i. and layered on polylysinated slides. Then, samples were fixed with 4% paraformaldehyde (PFA 4%), washed three times and stained with Hoechst 33342. Evan’s blue was used as a cell counter-stain. Samples were analyzed on a fluorescence microscope (Leitz, Wetzlar, Germany).

### 2.13. Statistical Analysis

The statistical differences between several sample types were analyzed with one-way analysis of variance (ANOVA) using Prism software (GraphPad). Asterisks (*, ** and ***) indicate the significance of *p*-values less than 0.05, 0.01 and 0.001, respectively.

## 3. Results

### 3.1. Total Phenol Content and Radical Scavenging Activity of NS and BS Extracts

The total phenol content and radical scavenging activity of NS and BS extracts are reported in Table 1.

In agreement with total phenolic content, NS showed the highest radical scavenging activity. Although the total phenolic content and the amount of polyphenols identified by HPLC in NS were similar to our previous investigation [2], the radical scavenging activity is nearly 10-times stronger with the current extract. This trend reflects the amount of phenols present in the almond skins, as well as the different phenolic profile of the samples (Table 2 and Table 3).

**Figure 1:**
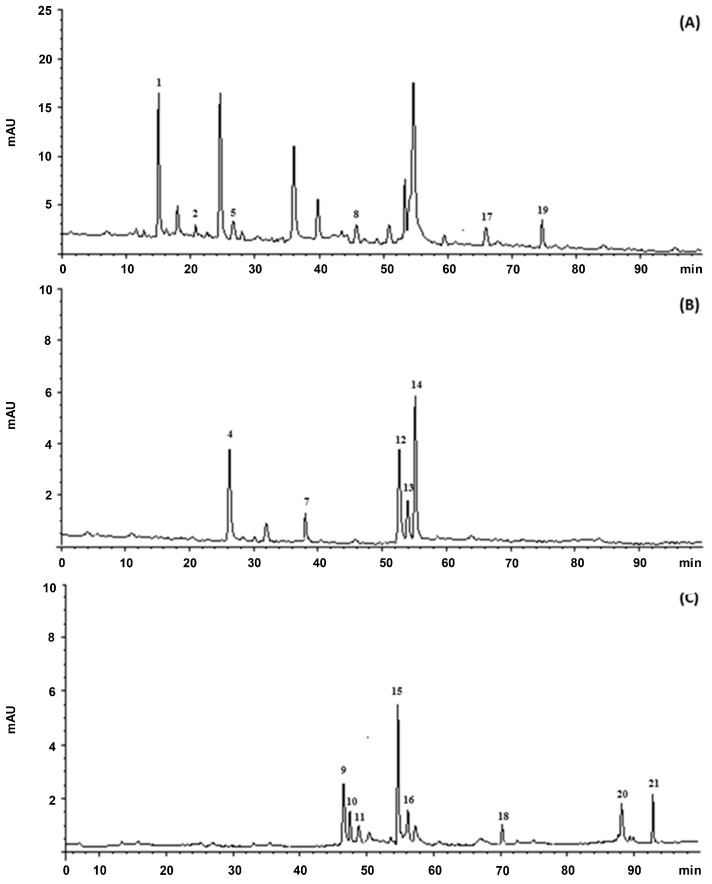

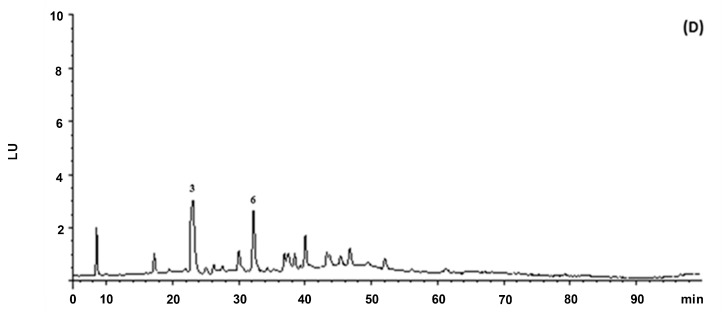
Representative HPLC chromatogram of phenolic compounds present in the methanol extract of NS: (**A**) 280 nm; (**B**) 330 nm; (**C**) 370 nm and (**D**) λ_ex_ 276 nm, λ_em_ 316 nm. The numbering of the peaks refers to Table 2.

Catechin and epicatechin appeared at much higher concentrations in the present extract. It is well known that a number of factors, including variety, cultivation and environmental conditions, as well as extraction methods have a significant impact on the polyphenolic composition of the extracts [26] (Figure 1).

### 3.2. Cytotoxicity of Almond Extracts on Cells Cultures

**Figure 2:**
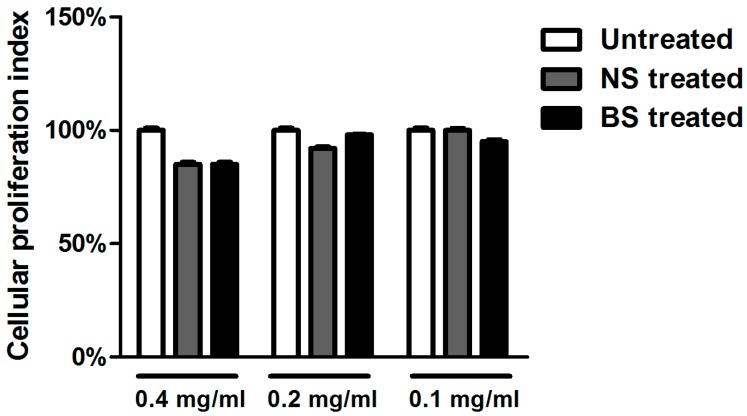
Viability assay in Vero cells treated with natural and blanched skin extracts. The cell viability was determined on the basis of ATP levels using the ViaLight^™^ plus cell proliferation and cytotoxicity bioassay kit (Lonza Group Ltd., Basel, Switzerland). Vero cells were treated with three different concentrations of NS and BS almond extracts (0.4 mg/mL, 0.2 mg/mL and 0.1 mg/mL). 24 h post-treatment, they were collected, and the luminescence value was converted into the cell proliferation index (%) as described in the Materials and Methods. The assay was performed as the means of triplicates ± SD.

To examine the cytotoxicity effect of NS and BS extracts, we incubated Vero cells in the presence of different concentrations of almond extracts for 24 h. Samples were then collected, and the quantification of the emitted light intensity, related to ATP degradation as a cellular proliferation index, was measured. The results showed that treatment with NS and BS extracts at the concentrations of 0.4, 0.2 and 0.1 mg/mL did not exhibit cytotoxicity (Figure 2).

### 3.3. Antiviral Activity of the Almond Extracts

**Figure 3:**
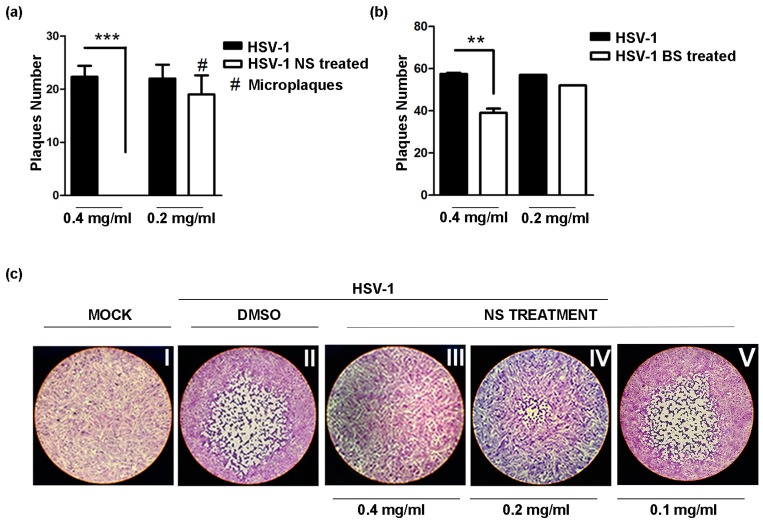
Plaque reduction assay to verify the antiviral activity of Californian natural and blanched skin almond extracts: Vero cells were infected with HSV-1 (F) and incubated for 1 h at 37 °C. After the incubation time, the inoculum was removed, and the monolayers were overlaid with Dulbecco’s Modified Eagle’s Medium containing 0.8% methylcellulose in the presence of NS and BS extracts at different concentrations (0.4 mg/mL, 0.2 mg/mL and 0.1 mg/mL). The plates were incubated at 37 °C and 5% CO_2_ for three days, and the plaques were visualized by staining cells with crystal violet. The DMSO was used in the HSV-1 control. Results are the mean ± SD of triplicate experiments and asterisks (** and ***) indicate significant changes of *p*-value less than 0.01 and 0.001, respectively. In (**a**,**b**), the plaque reduction assay was performed following NS and BS almond extracts treatment, respectively; (**c**) shows the plaque morphological change due to NS treatment 0.4 mg/mL vs. 0.2 mg/mL. No reduction was observed at 0.1 mg/mL.

To further investigate whether treatment with NS and BS interferes with viral replication, the plaque reduction assay was performed as described in the Materials and Methods. The results of standard plaque reduction assay showed a significant decrease in the viral titer after incubation with NS almond extract at the concentration of 0.4 mg/mL (*** *p* < 0.001) (Figure 3a). At the concentration of 0.2 and 0.1 mg/mL, NS did not show a relevant viral titer reduction; however, a decrease in the size of plaques (micro-plaques) was observed at 0.2 mg/mL (Figure 3c, IV). BS treatment showed a viral titer reduction at 0.4 mg/mL (** *p* < 0.01) only, but not at 0.2 or 0.1 mg/mL. Therefore, these results suggest a direct association between increasing concentration of the NS almond extract and the antiviral activity. Indeed, the increased concentration of the NS extract was accompanied by a total reduction of the virus replication.

### 3.4. Inhibition of HSV Replication by NS and BS Extracts

**Figure 4:**
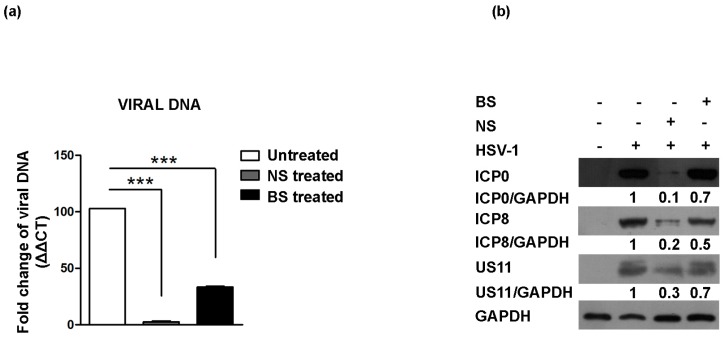
Effect of almond extracts treatment on the HSV-1 replication and on viral antigens’ expression. (**a**) The viral DNA was extracted from Vero cells 24 h post-HSV-1 infection as described in the Materials and Methods. Relative quantization of viral DNA was performed using real-time quantitative PCR and analyzed by the comparative *C*_t_ method (ΔΔ*C*_t_). Values represent ±SD of the average of three samples normalized against the GAPDH copies number; (**b**) Immunoblot analysis was performed in Vero cells HSV-1 infected and treated with natural (NS) and blanched (BS) skin extracts (0.4 mg/mL). Equal amounts of proteins were separated by polyacrylamide gel electrophoresis, transferred and probed with specific antibody to α (ICP0), β (ICP8) and γ (US11) viral proteins. GAPDH proteins were used for housekeeping. Band density was determined with the T.I.N.A. program and was expressed as the fold change over the appropriate housekeeping genes. Asterisks (***) indicate significant changes of *p*-values less than 0.001.

To determine whether treatment with the almond extracts interferes with active viral replication, two different approaches were enrolled: (i) detecting viral DNA by using real-time PCR; (ii) detecting viral proteins cascade by Western blot analysis. First of all, the different levels of total viral DNA were analyzed following NS and BS extract treatment compared to untreated HSV-1 at 24 h p.i. The results showed that NS and BS almond extracts at the concentration of 0.4 mg/mL were able to block viral DNA accumulation, to different degrees, when compared to the untreated infected cells (*** *p* < 0.001) (** *p* < 0.01), respectively (Figure 4a). Inhibition of viral replication also was measured by quantification of viral protein expression of representative α ICP0, β ICP8 and γ Us11 viral proteins. The viral proteins chosen are representative genes of the three classes of viral genes transcribed in an ordered cascade. Therefore, immunoblot analysis was performed in Vero cells infected with HSV-1, either treated or not with NS and BS extracts separately and incubated for 24 h. Data demonstrated that the accumulation of ICP0, ICP8 and Us11 viral proteins was considerably reduced in NS-treated infected cells if compared to the untreated infected cells. Infected cells treated with BS displayed less decrease of all three different viral proteins, confirming the data obtained by viral DNA quantification (Figure 4b). Western blot band intensities of the treated or not infected cells were normalized with respect to the untreated HSV-1 infected control using T.I.N.A. software (version 2.10, Raytest, Straubenhardt, Germany). 

### 3.5. HSV Attachment to Target Cells Is Prevented by Almond Extracts

**Figure 5:**
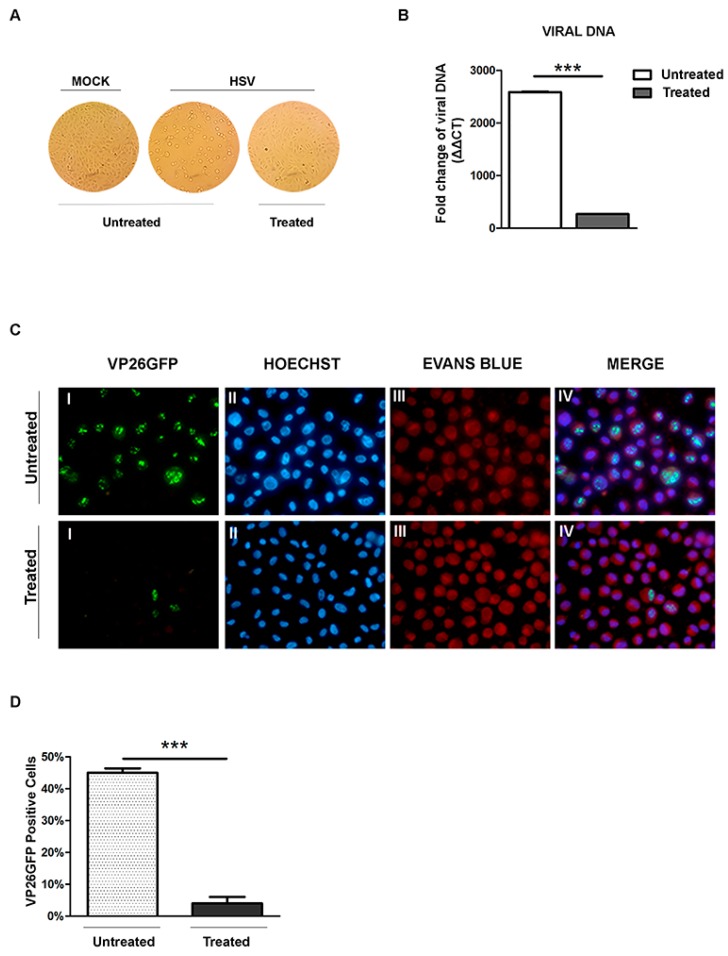
Effect of almond extract treatment on the HSV-1 binding. Vero cells infected and mock-infected with HSV-1 at low temperature and processed as described in the Material and Methods. (**A**) Normal phase contrast inverted micrographs of processed samples are shown; (**B**) Relative quantization of viral DNA of the processed samples are shown. Values represent ±SD of the average of three samples normalized against the GAPDH copies number and were analyzed by the comparative *C*_t_ method (ΔΔ*C*_t_); (**C**) Fluorescent images of Vero cells infected with VP26GFP HSV-1 virus and following virus inactivation, untreated and treated with NS extract. The green dots (I) represent VP16GFP viral antigen localization. The cells were stained with Hoechst (II) for the nuclear compartment and Evans blue dye (III) as a counter-stain. The VP26GFP, Hoechst and Evans blue images are merged in the last columns (IV); (**D**) Schematic representation of the VP26GFP-positive cells. Asterisks (***) indicate significant changes of *p*-values less than 0.001.

Since the 0.4 mg/mL concentration of NS resulted in a >90% decrease of viral titer and was found to be non-toxic to Vero cells, we focused on the 0.4 mg/mL concentration of NS in subsequent experiments. Indeed, to better understanding the molecular mechanisms mediated by NS almond extracts on HSV-1 replication, we examined whether NS affected the capability of HSV-1 to bind to the cellular membrane. The earliest stage of the HSV infection is the binding to the cellular membrane responsible for the release of core and tegument proteins into the cytoplasmatic compartment. The above data were obtained by NS treatment of infected cells after viral inoculum. Based on these considerations, a binding assay was carried out to allow the virus to bind to cellular receptors, but not to enter the cells. Attached viral particles can later enter cells after incubation at 37 °C, and they can complete the lytic cycle. Therefore, viral suspensions were incubated or not with NS (0.4 mg/mL), and infection was carried out in ice as reported in Section 2. The samples were subjected to: (i) live detection of cytopathic effect (CPE); (ii) real-time PCR separately, to investigate whether NS treatment prevents the viral attachment to a host membrane. The results shown in Figure 5A indicated that NS was able to inhibit the infectivity; indeed no cytopathic effect was visualized by inverted microscopy in the treated infected cells. Moreover, quantification of viral DNA by real-time PCR demonstrated that incubation of the viral suspension at low temperature with NS extracts efficiently inhibits viral DNA accumulation (*** *p* < 0.001) if compared to the infected untreated cells (Figure 5B). To confirm these data, we employed a recombinant virus able to express a structural VP26 tagged with green fluorescent protein (GFP). VP26GFP-HSV-1 viral suspensions were subjected to the binding assay and used to infect Vero cells. Figure 5C demonstrated the accumulation of the auto-fluorescence of viral protein VP26 during the late stage of viral replication only in less 5% of the treated infected cells if compared to the untreated infected cells. Indeed, in the untreated-infected green dots corresponding to VP26 viral protein accumulation, VP26GFP-positive cells were observed. In the treated-infected cells, the green dots decreased significantly (Figure 5C, I).

## 4. Discussion

Frequent HSV outbreaks can have major psychological and social impacts on infected individuals. Further, HSV lesions provide an easy route for HIV infection during sexual activity [27]. Therefore, new medications are required to block infection and to prevent viral shedding by an infected individual. Approximately half of the drugs currently in clinical use are of natural product origin [28]. Beyond the strong antioxidant activity, it is interesting to note that the same polyphenols have already shown antiviral activity against several strains. Kaempferol showed a selectively anti-influenza B virus activity due to a more planar flavonol structure with only one C-4′ phenolic hydroxyl group in the B ring, which appears to be essential to carry out these activities [29]. Quercetin was found to significantly reduce the replication of influenza viruses in vitro and in vivo, and in vitro, anti-Mayaro virus [30,31,32]. Amongst the polyphenols mostly identified in the current extract, epicatechin was the strongest one, possessing the lowest EC_50_ among polyphenols tested in the DPPH assay, followed by quercetin, catechin, protocatechuic acid and kaempferol [33]. The *O*-dihydroxy group on ring B (catechol) plays a crucial role in radical scavenging activity in the DPPH assay; in fact, the weaker antiradical activity of kaempferol and naringenin-7-*O*-glucoside could be ascribed to the absence of this. Furthermore, the stronger antiradical activity of flavonoids could be ascribed to the presence of other key elements, such as 2–3 double bond conjugated with the 4-oxo function and hydroxyl groups in the 3 and/or 5 position [34]. Amongst phenolic acids, those with two hydroxyl groups bonded to the aromatic ring in the ortho position, like protocatechuic acid, showed strong antioxidant and anti-radical activity in the DPPH assay [35]. In light of this, we can affirm that all more prevalent compounds in the current extract contribute to the strongest antioxidant activity found, but the authors agree that this activity is attributable in particular to the high concentration of quercetin, epicatechin and catechin, flavonoids widely investigated, which showed the strongest antioxidant and anti-radical activity in the DPPH assay. The present study indicates that quercetin, the most abundant compound identified in the current extract, was found to be virucidal against HSV-1. It has been reported that anthocyanins, chlorogenic acid, quercetin and kaempferol are very good scavengers of reactive oxygen and nitrogen, including the hydroxyl radical and, therefore, perfectly protect lipids against peroxidation [36]. A major and very important place of attack by free radicals in the organism is the cell membrane. Oxidation of its components and, in particular, the membrane lipids by free radicals causes structural changes, interfering with membrane functions, which lead to pathological changes in the human organism. Hemolytic tests indicate that compounds present in the tested extracts do not penetrate deep into the hydrophobic part of the membrane [37]. Data presented here indicate that both BS and NS extracts did not display cytotoxicity towards Vero cells at all concentrations tested and exerted effect on HSV-1 in a dose-dependent manner. However, parental HSV-1 DNA persisted after infection of Vero cells following NS treatment serving as an effective PCR template for HSV-1 genes (Figure 4a). These data suggested the capability of viral particles to enter into the cells, but the addition of almond skin extracts after 1 h post-infection blocks the virus spread to neighboring cells, thus justifying the absence of detectable plaques (Figure 3). On the other hand, the treatment of virions for one hour with 0.4 mg/mL of NS at 4 °C and the subsequent absorption of viral inoculum caused a fall in infectivity, as measured by ECP and viral DNA quantification (Figure 5). Indeed, the removal of the NS extracts after one hour of viral adsorption resulted in a significant neutralization of the virus in Vero cells. One potential mechanism for this inhibitory effect of NS on the HSV-1 lytic cycle is by blocking virion entry into the cells. Literature data have shown an effect of flavonones, such as EGCG, on HIV and influenza virus infection [38,39]. Alternatively, the viral particle integrity could be damaged, as has been previously observed for the effect of flavonones on HSV-1 [40]. Indeed, the virion consists of three major structures, an outer portion called the “envelope”, which includes more than 11 glycoproteins, a tegument layer composed of 15 proteins and an icosahedral capsid enclosing the viral DNA [41,42,43].

## 5. Conclusions

This study demonstrated that almond skin extracts exhibit an antiviral activity blocking HSV-1 infection. Indeed, they exert a dual action by restricting virus inside the cells and by hampering the virus absorption after exposition to a high content of polyphenols present in almond skin. Therefore, the results presented in this study indicated flavonones as an attractive candidate for the development of novel natural virucide compounds for the prevention of HSV infections. Finally, we believe that this extraction process could add value to the almond biomass for the valorization of food processing wastes.

## Figures and Tables

**Table 1:** Total phenol content and radical scavenging activity in natural (NS) and blanched (BS) almond skins.

Sample	(mg of Skin *)	mgGAE/100 g FW
NS	0.032 ± 0.001	4228.52 ± 265.19
BS	0.117 ± 0.003	3841.16 ± 18.56

* Activity is expressed as SE_50_ (amount needed to scavenge 50 µmoles of the initial DPPH^•^ solution).

**Table 2:** Polyphenolic compounds identified in natural (NS) and blanched (BS) almond skins.

Peak No.	Compound	* RT (min)	λ_max_ (nm)
1	Protocatechuic acid	13.081	259; 294
2	*p*-Hydroxybenzoic acid	20.442	255
3	Catechin	23.616	278
4	Chlorogenic acid	26.510	296; 326
5	Vanillic acid	26.892	261; 292
6	Epicatechin	32.561	278
7	*trans*-*p*-Coumaric acid	38.015	310
8	Eryodictiol-7-*O*-glucoside	45.933	284
9	Quercetin-3-*O*-rutinoside	47.434	256; 354
10	Quercetin-3-*O*-galactoside	47.757	256; 354
11	Quercetin-3-*O*-glucoside	48.892	256; 354
12	Kaempferol-3-*O*-rutinoside	53.395	265; 347
13	Kaempferol-3-*O*-glucoside	54.279	265; 347
14	Naringenin-7-*O*-glucoside	54.555	284; 338
15	Isorhamnetin-3-*O*-rutinoside	54.937	256; 354
16	Isorhamnetin-3-*O*-glucoside	56.650	254; 354
17	Eriodictyol	66.052	288
18	Quercetin	70.367	256; 372
19	Naringenin	73.018	288
20	Kaempferol	88.516	264; 366
21	Isorhamnetin	93.060	254; 370

Bold values correspond to the peak number present in the chromatograms. * RT = Retention time.

**Table 3:** Polyphenolic compounds in natural (NS) and blanched (BS) almond skins. Values are expressed as µg/100 g and represent the average (±SD) of three independent experiments (*n* = 3).

Compound	NS	BS
*Hydroxybenzoic acids*		
Protocatechuic acid	3862.97 ± 124.52	878.24 ± 22.65
*p*-Hydroxybenzoic acid	7094.27 ± 224.10	696.21 ± 32.41
Vanillic acid	5652.08 ± 236.54	709.027 ± 11.35
*Hydroxycinnamic acids*		
Chlorogenic acid	1144.54 ± 84.25	375.41 ± 8.54
*trans*-*p*-Coumaric acid	791.62 ± 22.45	142.19 ± 5.24
*Flavanones*		
Eriodictyol	2777.23 ± 78.65	364.8 ± 12.54
Eryodictiol-7-*O*-glucoside	57.9 ± 1.26	8.33 ± 0.221
Naringenin	5802.51 ± 185.44	474.54 ± 20.85
Naringenin-7-*O*-glucoside	34,020.11 ± 654.22	2148.22 ± 62.35
*Flavonols*		
Kaempferol-3-*O*-rutinoside	8062.45 ± 261.33	1037.64 ± 52.14
Kaempferol-3-*O*-glucoside	29,530.44 ± 854.22	522.84 ± 21.47
Isorhamnetin-3-*O*-glucoside	16,597.37 ± 546.31	908.86 ± 18.95
Quercetin	3474.03 ± 105.62	2035.8 ± 68.54
Kaempferol	5573.6 ± 88.65	998.84 ± 22.64
Isorhamnetin	3513.47 ± 112.35	230.4 ± 9.87
Quercetin-3-*O*-rutinoside	1067 ± 55.62	299.55 ± 8.03
Quercetin-3-*O*-galactoside	1400.8 ± 37.45	123.56 ± 4.42
Quercetin-3-*O*-glucoside	1219.53 ± 24.89	108.67 ± 2.89
Isorhamnetin-3-*O*-rutinoside	17,620.45 ± 495.65	1526.53 ± 23.98
*Flavanols*		
Epicatechin	26,948.72 ± 554.25	1900.8 ± 62.74
Catechin	47,998.32 ± 956.54	4210.91 ± 88.54
Total amount	224,209.41	19,701.36
